# An Exploration into Diffusion Tensor Imaging in the Bovine Ocular Lens

**DOI:** 10.3389/fphys.2013.00033

**Published:** 2013-03-01

**Authors:** Ehsan Vaghefi, Paul J. Donaldson

**Affiliations:** ^1^Auckland Bioengineering Institute, University of AucklandAuckland, New Zealand; ^2^Department of Optometry and Vision Sciences, University of AucklandAuckland, New Zealand

**Keywords:** ocular lens, MRI, diffusion tensor imaging, fractional anisotropy, micro-circulation

## Abstract

We describe our development of the diffusion tensor imaging modality for the bovine ocular lens. Diffusion gradients were added to a spin-echo pulse sequence and the relevant parameters of the sequence were refined to achieve good diffusion weighting in the lens tissue, which demonstrated heterogeneous regions of diffusive signal attenuation. Decay curves for *b*-value (loosely summarizes the strength of diffusion weighting) and *T*_E_ (determines the amount of magnetic resonance imaging-obtained signal) were used to estimate apparent diffusion coefficients (ADC) and *T*_2_ in different lens regions. The ADCs varied by over an order of magnitude and revealed diffusive anisotropy in the lens. Up to 30 diffusion gradient directions, and 8 signal acquisition averages, were applied to lenses in culture in order to improve maps of diffusion tensor eigenvalues, equivalent to ADC, across the lens. From these maps, fractional anisotropy maps were calculated and compared to known spatial distributions of anisotropic molecular fluxes in the lens. This comparison suggested new hypotheses and experiments to quantitatively assess models of circulation in the avascular lens.

## Introduction

**Figure 1:**
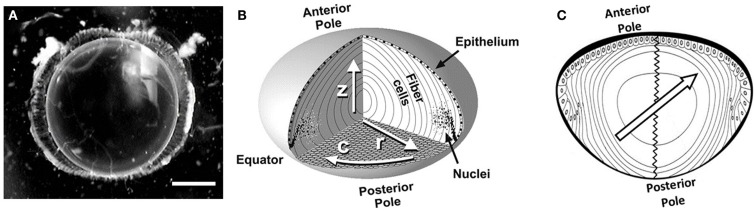
**The ocular lens**. **(A)** A clear crystalline rat lens with visual axis oriented toward the viewer. Scale bar, 1 mm. **(B)** A simplified diagram of the lens cellular structure showing anterior and posterior poles; inner, elongated fiber cells; and an outer layer of cuboidal epithelial cells over the anterior side of the lens. Fiber cell nuclei degrade as the cells age (toward the lens center). Arrows label directions within the lens as follows: *r*, radial; *c*, circumferential; *z*, visual axis. Reproduced with permission from Jacobs et al. (2004). **(C)** The 2D schematic of the lens axial cut with the anterior and posterior poles marked and the zigzag sutures connecting the poles illustrated. The center-located arrow demonstrates the direction of the applied excitation gradient, which is in parallel with the cortical fiber cell directions in upper-left and lower-right regions. At the same time it is perpendicular to the fiber cell extrusion direction in the upper right and lower left sections.

The ocular lens appears deceptively simple: like a transparent glass element of an engineered optical device such as a camera lens. In reality, the ocular lens is a complex assembly, mainly of elongated fiber cells which confer transparency and allow focal accommodation, by maintaining tightly controlled cellular biochemistry, volume regulation, and structural integrity (Figure 1; Koretz and Handelman, 1983; Pierscionek and Chan, 1989; Donaldson et al., 2001; Kuszak et al., 2006; Davidovits, 2008).

Lens transparency must be constantly upheld by homeostasis within the tissue; but it also requires an absence of blood vessels in the light path (Mathias et al., 1981; Baldo and Mathias, 1992). This paradox of homeostasis in the absence of blood circulation appears to be solved in the lens by an avascular micro-circulation system, capable of supplying oxygen and nutrients throughout the lentoid mass of cells, via cell membrane pumps, transporters and channels, and extracellular circulatory routes (Mathias et al., 1997, 2010). Through such mechanisms, cells of the lens appear to maintain the overall osmotic balance and structural integrity of the organ.

Data supporting a variety of avascular fluxes in the lens have been obtained by direct and indirect probing techniques such as electrophysiological impedance analysis; dye tracer studies; pharmacological perturbation experiments; and transgenic reporter and mutation studies (Robinson and Patterson, 1982; Bassnett et al., 1994; Rae et al., 1996; Shestopalov and Bassnett, 2000; Alvarez et al., 2003; Wang et al., 2009). While this variety of studies has made great progress toward understanding the biophysical basis of ocular lens circulation and transparency, there remains a need for direct, non-invasive study of micro-circulation in the unperturbed lens, and particularly for interrogation deep within the lens tissue.

The goal of non-invasive interrogation deep within the lens has been technically challenging due to the size and shape of the fiber cell mass which, depending on the species, can range from millimeters to centimeters in radius while having an almost spherical aspect ratio (Kuszak et al., 2004a). This geometry makes the center of the lens largely inaccessible by non-destructive techniques.

A promising strategy for addressing this challenge was suggested by early studies which applied the technique of magnetic resonance imaging (MRI) to probing biophysical aspects of the ocular lens (e.g., Ahn et al., 1989; Moffat et al., 1999; Lizak et al., 2000). Since MRI is a non-invasive imaging modality capable of deep interrogation in biological subjects, these studies were able to effectively probe the intact lens to its center. The studies developed the first methods for using MRI to characterize intrinsic lens properties such as water content, magnetic resonance relaxation times, and patterns of apparent water diffusivity as shown by diffusion tensor analysis (Moffat and Pope, 2002a). More recently, Vaghefi et al. (2009) performed an initial study of diffusivity using another method of diffusion tensor imaging (DTI; Basser et al., 1994; Le Bihan et al., 2001) in the bovine lens. The DTI modality characterizes a tensor distribution describing the apparent diffusion coefficients (ADC) for the self-diffusion of water throughout the subject. This study revealed further aspects of lens diffusivity, in particular a map of diffusivity eigenvectors and associated ADCs, which showed anisotropic water movements in the outer cortex and isotropic patterns in the core of the lens.

In this paper, we report on our broader program to characterize a range of diffusion-weighted MRI regimes in the ocular lens, and our subsequent development of a superior DTI protocol for the lens. We describe our empirical and theoretical rationale, and novel quantitative results which have followed from these studies. Our results demonstrate the efficacy of DTI in the ocular lens, and the utility of this modality for developing new hypotheses for lens circulation mechanisms. We envisage that the DTI modality will provide new avenues for non-invasive studies of ocular lens physiology, with possible applications in clinical diagnostics or therapeutics.

## Materials and Methods

All animals used in this study were treated in accordance with institutional guidelines and the ARVO Resolution on the Use of Animals in Research. Chemicals were obtained from Sigma-Aldrich New Zealand. Bovine eyeballs were collected from animals immediately after slaughter at Auckland Abattoir (New Zealand) and transferred into phosphate buffered saline (PBS: NaCl 137 mM; KCl 2.7 mM; Na_2_HPO_4_ 10 mM; KH_2_PO_4_ 1.76 mM; pH 7.4). Following transport to The University of Auckland, and within 2 h of death, ocular lenses were extracted and transferred to artificial aqueous humor solution (AAH: NaCl 130 mM; KCl 5 mM; MgCl_2_ 0.5 mM; CaCl_2_ 1 mM; NaHCO_3_ 10 mM; glucose 5 mM; buffered with 10 mM HEPES to pH 7.1) at 35°C. The osmolality of this medium was adjusted to 300 mOsm/kg by adding sucrose.

All MRI experiments were performed at the small animal MRI facility, Centre for Advanced MRI, The University of Auckland, which was comprised of a Varian Unity Inova 4.7 T horizontal bore MRI unit (Varian Inc., Palo Alto, CA, USA), with a 65 mm ID, 100 G/cm gradient system. Images were acquired using a 40 mm ID RF probe based on Varian’s Millipede™ design. The temperature inside the scanner bore was monitored and controlled so that the lenses and the AAH solution were maintained at 35°C throughout the experiments. The sample holder was designed using poly-acrylic plastic to aid in maintaining a constant temperature and allow an efficient arrangement of samples inside the RF coil.

To observe the signal properties and resolution in the lens without any diffusion weighting applied, *T*_2_-weighted images of a single slice of the lens were acquired using a spin-echo pulse sequence with a 32 mm × 32 mm FOV, 64 × 64 acquisition matrix, slice thickness = 0.5 mm, TR = 2000 ms and *T*_E_ = 12 ms. The slice was positioned to pass through the central axis of the lens along the normal light path (the visual axis). These settings were used for all the replications in our series.

Additional gradients were subsequently applied in the spin-echo pulse sequence to obtain diffusion weighting. Parameters governing the acquired signal were then varied systematically, within the constraints of our MRI hardware and lens culture system, in order to achieve the best DTI results.

**Figure 2:**
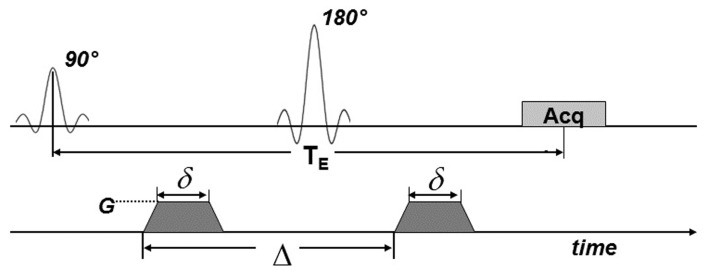
**MRI pulse sequence for diffusion weighting**. Diffusion gradients (dark shading) were applied on both sides of the 180° pulse. Here, *T*_E_ is the echo time; Acq indicates the signal acquisition block. *G* is the amplitude of the diffusion gradients, δ is their duration and Δ is the delay time between them.

To obtain diffusion-weighted images, a bi-polar gradient was added on both sides of the 180° pulse of the spin-echo sequence, as shown in Figure 2. Using this pulse sequence, the amount of signal attenuation is determined by Eq. 1 (Stejskal, 1965; Jones et al., 1999).

(1) $$S=S_{0}e^{-T_{E}∕T_{2}}e^{-b{\vec{g}}^{T}D\vec{g}}$$

where

$$b=\gamma^{2}G^{2}\delta^{2}\left(\Delta -\frac{\delta }{3}\right)$$

Here *S* is the observed signal intensity, *S*_0_ the maximum signal intensity without relaxation or diffusion effects; *e* is the natural logarithm base, *T*_E_ the echo time; $\vec{g}$ is the normalized diffusion gradient vector, *D* is the diffusion tensor matrix; γ is the gyromagnetic ratio, *G* the magnitude of the diffusion weighting gradients; δ is the gradient duration and Δ the delay time between the diffusion weighting gradients. Where only one diffusion gradient direction is applied, *D* is a scalar term and $\vec{g}$ is omitted.

Diffusion-weighted images were acquired with FOV, acquisition matrix, slice thickness, and TR as in the spin-echo sequence. *T*_E_ and *b*-value were varied as described below to determine the appropriate parameter values. For these experiments the slice was again positioned to pass through the visual axis of the lens and the diffusion gradients were applied in one direction, at an angle of ∼45° to this axis. This specific gradient direction was chosen due to the symmetry of the lens cellular structure (Figure 1B). Using this orientation, the excitation gradient would be in parallel with the cellular structure of some regions of the cortex, while is perpendicular to the fiber extrusion direction of the other regions (see Figure 1C). This deliberate diffusion gradient direction planning generates the possibility of studying radial and circumferential diffusion coefficients in the lens cortex simultaneously and performing a single scan.

The diffusion-weighted signal behavior in the bovine lens was explored for a range of *T*_E_ and *b*-values. In the first set of experiments, the *T*_E_ value was kept constant while the *b*-value was varied between 214 and 2264 s/mm^2^, in steps of roughly 200 s/mm^2^. The *T*_E_ in these experiments was set as short as possible given our hardware (12 ms); this was motivated by the known, short *T*_2_ of the ocular lens (see below). To achieve this, the δ and Δ values were fixed at 3.3 and 6.6 ms respectively. The range of *b*-values was generated by varying *G* from 20 to 65 G/cm. This routine was applied to two replicate lenses.

In the second set of diffusion-weighted experiments, the merit of using a short *T*_E_ was tested. Diffusion-weighted images were acquired for four TEs (12, 17, 22, and 30 ms) combined with four different *b*-values (1320, 1392, 1467, and 1543 s/mm^2^) which spanned a range selected from the first set of experiments (see Results). For each *T*_E_, the parameters δ and Δ were kept constant at the values indicated above; the four different *b*-values at each *T*_E_ resulted from adjusting *G* between 37 and 40 G/cm. This routine was also applied to two replicate lenses.

Using the *b*-value and *T*_E_ that were finally chosen from these experiments (*b* = 1320 s/mm^2^, *T*_E_ = 12 ms; see Results), the effects of multiple diffusion gradient directions and signal averaging on DTI were then investigated. The combinations of diffusion gradient directions and signal averages tested in these experiments are shown in Table 1. The total time for each experiment was restricted to ∼10–12 h in order to stay well within an observed 20 h limit, after which the cultured bovine lenses showed initial signs of cataracts indicating loss of homeostasis. Working within this time limit, there was a necessary trade-off between the number of diffusion gradient directions and the number of signal averages possible given our hardware. Nevertheless, it was feasible to evaluate up to 30 directions and up to 8 signal averages within 12 h. The experiments in Table 1 were replicated as listed there and using the general parameters (e.g., slice orientation, thickness, etc.) listed above.

**Table 1:** **A series of DTI experiments performed on the bovine lens to evaluate the effects of gradient directions and signal averaging**.

Number of diffusion gradient directions	Number of averages	Scan duration (h:min)	Number of lens replicates
6 directions	8	9:50	10
12 directions	4	10:35	6
20 directions	2	11:50	5
30 directions	1	10:55	5

*The number of diffusion gradient directions; signal averages; and the total scan times required for the different regimes are shown, with the number of replicate lenses imaged in each experiment*.

The DTI post-processing was done using the open-source 3D Slicer software package (Pieper et al., 2004). Further image post-processing and analysis were performed using custom-written routines in the MATLAB array-oriented scripting language (The MathWorks Inc., Natick, MA, USA). In order to extract values for *T*_2_ and *D* from diffusion-weighted images (see Results; Figures 5 and 7), datasets were fitted with exponential curves (Eq. 1). Curve-fitting was performed using the MATLAB toolbox, EZYFIT (downloaded from http://www.fast.u-psud.fr/ezyfit/). A recursive process using unconstrained non-linear minimization of the sum of squared residuals was used (Moisy, 2009).

## Results

In this study we developed DTI for application in the ocular lens. By considering the empirical performance of various DTI parameter combinations, we were able to design a protocol for DTI in the bovine lens. This protocol showed the potential of the DTI modality to reveal diffusive constraints imposed by the lenticular tissue, with possible implications for the micro-circulatory properties of this organ.

**Figure 3:**
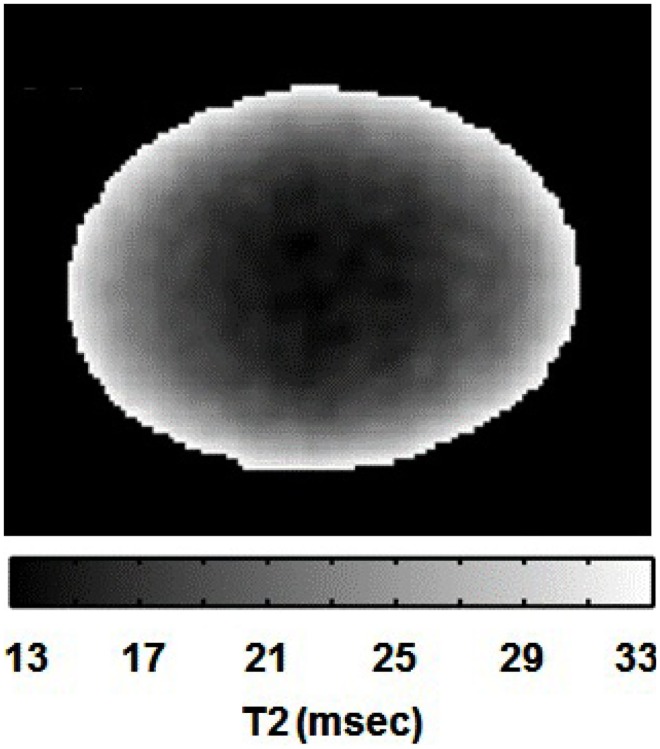
***T*_2_ gray-scale image of the bovine lens**. The lightest area has the longest *T*_2_ values and the darkest area has the shortest *T*_2_ values.

As an initial step, we acquired *T*_2_-weighted images of the lens using a spin-echo sequence, to evaluate the signal behavior without any diffusion weighting. As the *T*_2_ relaxation times of the ocular lens are known to be relatively short (Patz et al., 2007), the short *T*_E_ used here (12 ms) was deemed appropriate for *T*_2_ weighting. A *T*_2_-weighted image of the bovine lens, acquired using our protocol, is shown in Figure 3. The lens was clearly visible, with the shading increasingly dark toward the lens center as a result of shorter *T*_2_ values. This decrease in *T*_2_ within the lens was consistent with data from the literature (Cheng et al., 1987; Moffat and Pope, 2002b) indicating increasing protein concentration toward the center of the ocular lens.

**Figure 4:**
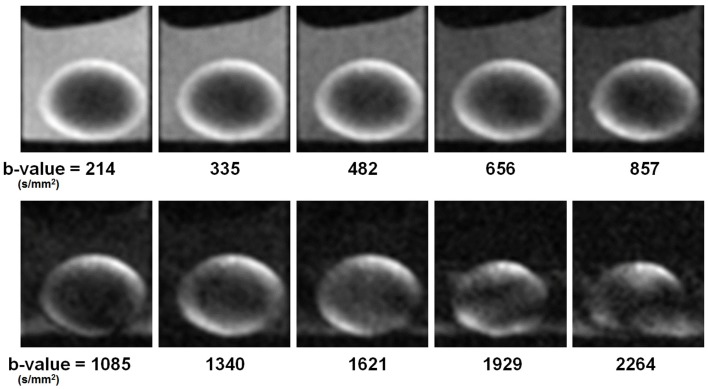
**Ocular lens imaged using a diffusion weighting pulse sequence, varying *b*-value**. Ten different *b*-values were tested in the first set of diffusion-weighted experiments (see Materials and Methods). Image slices were positioned to pass through the visual axis of the lens (see Figures 1B,C). The direction of the diffusion gradients (see Figure 2) was from bottom-left to top-right in the plane of the images. Improvement in the diffusion weightings of the images appeared as greater signal differentiation around the lens cortex, with diffusive attenuation in the upper-left and lower-right regions visibly stronger as *b*-value was increased from 214 to 1340 s/mm^2^. Further increase of the *b*-value resulted in imaging artifacts (see Text). Lenses were oriented with anterior pole facing down and posterior pole facing up.

To obtain diffusion-weighted images, a bi-polar gradient was added on both sides of the 180° pulse of the spin-echo sequence (see Materials and Methods; Figure 2). In the first set of experiments, the effects of the *b*-value on diffusion weighting (see Eq. 1) were investigated. The *T*_E_ was kept as short as possible (again 12 ms) while the *b*-value was varied from 214 to 2264 s/mm^2^. Selected images resulting from the diffusion-weighted scans are shown in Figure 4. In these images it was apparent that with increasing *b*-value, the signal in the upper-left and lower-right areas of the lens cortex was attenuated as expected from Eq. 1. This attenuation was especially visible at *b*-values > 600 s/mm^2^. Since the direction of the diffusion gradients was from bottom-left to top-right in these images (i.e., at ∼45° to the visual axis; see Materials and Methods), the observed attenuation suggested measurable diffusivity in this direction. Toward the center of the lens the overall signal decreased. At *b*-values > 1620 s/mm^2^ however, significant blurring artifacts were observed, possibly due to vibration from rapid switching of large magnetic field gradients, as has been reported in other studies (e.g., Gallichan et al., 2010).

**Figure 5:**
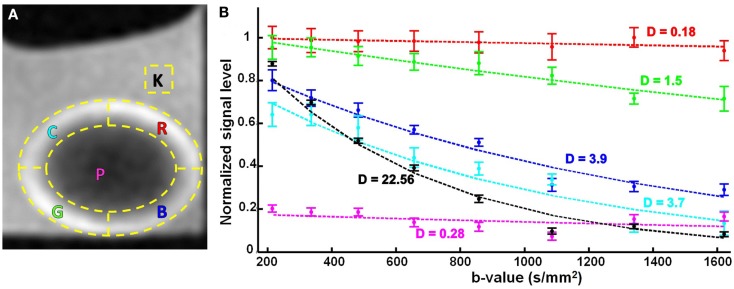
**The effects of varying *b*-value, on the diffusion-weighted signal from the ocular lens**. **(A)** Six regions of interest (ROIs; yellow dashed lines) were selected in lens image slices from unidirectional diffusion-weighted scans (Figure 4). The direction of the diffusion gradients was from bottom-left to top-right in the plane of the images. The letter inside each ROI stands for the color of the corresponding signal plot in **(B)**, namely cyan (C), red (R), pink (P), green (G), blue (B), and black (K). Note, ROIs G and R sample areas where the direction of the diffusion gradients runs predominantly radially in the lens tissue (see Figure 1C); in contrast, ROIs B and C sample areas where the direction of the diffusion gradients runs more tangentially to the lens curvature. **(B)** Graphs of the mean and standard error of the signal measured in the respective color-coded ROIs for eight different *b*-values. The dashed lines represent exponential curves fitted to estimate the diffusion coefficients (labeled D near each curve) in the ROIs with respect to the applied diffusion gradient. The values for *D* are given in 10^−3^ mm^2^/s. The *T*_E_ in these experiments was 12 ms.

The attenuation observed in the diffusion-weighted series of images was quantified by segmenting the signal from six regions of interest (ROIs) which are outlined in Figure 5A. The locations of these ROIs with respect to the lens tissue, and the direction of the diffusion gradients from bottom-left to top-right in these images, were important. The images were oriented in a way that the lenses were positioned horizontally. The outer 30% of the lens radius was considered to be the cortex and the horizontal and vertical axes were then used to create five ROIs inside the lens. Two of the ROIs (labeled G and R in Figure 5A) formed circumferential arcs in the lens cortex that sampled areas where the direction of the diffusion gradients ran predominantly radially in the lens tissue (see also, Figures 1B,C). Two different ROIs (labeled B and C in Figure 5A) formed arcs in the cortex that sampled areas where, in contrast, the direction of the diffusion gradients was not radial in the lens but ran more tangentially to the lens curvature. The remaining ROIs defined a region from the lens core (labeled P); and a region from the bathing solution (AAH) outside the lens (labeled K).

The mean and standard error of the signal measured in each of these ROIs were graphed as a function of increasing *b*-value (Figure 5B; up to a *b*-value of 1621 s/mm^2^). The graph for the bathing solution outside the lens showed signal decreasing the most rapidly among all the ROIs, as expected from Eq. 1 for a freely diffusing solution. In contrast, the signal from the protein-rich lens core remained uniformly low with increasing *b*-value. In ROIs G and R, modest decreases of signal were observed with increasing *b*-value; while ROIs B and C showed similar, substantial signal attenuation as *b*-value was increased. Notably, for the *b*-values of 1085 s/mm^2^ and higher, the signal from the bathing solution was comparable to, or below, that of the lens core as a result of increasing diffusion weighting.

The data in Figure 5B showed that increasing the *b*-value allowed greater signal differentiation between the ROIs in the lens cortex. The greatest differentiation achieved between the ROIs while avoiding image artifacts, was observed at a *b*-value of 1340 s/mm^2^. Since image artifacts first became apparent at the next increment in *b*-value tested (*b* = 1621 s/mm^2^), we concluded from these experiments that *b*-values between ∼1300 and 1600 s/mm^2^ might be appropriate for studying the diffusion characteristics of the ocular lens, and should be evaluated further in the subsequent experiments (below).

Using the signal measurements from the ROIs, graphed in Figure 5B, it was possible to quantify the tissue diffusivity for water in each lens region. Exponential curves describing the signal attenuation as a function of *b*-value were fitted to the data from each ROI by adjusting *D* (Eq. 1) and the curves were plotted in Figure 5B, and labeled with the respective estimates of *D*. These estimates showed that diffusivity in the lens core was about 80-fold lower than in the aqueous bathing solution. The cortical ROIs B and C, which sampled areas where the direction of the diffusion gradients ran tangentially to the lens curvature, yielded values of *D* that were about 13-fold higher than in the lens core, and 6-fold lower than in the bathing solution. The cortical ROIs G and R, which sampled areas where the direction of the diffusion gradients was mainly radial in the lens cortex, yielded values of *D* whose average was 3-fold higher than in the lens core and 27-fold lower than in the bathing solution. These values showed that in ROIs B and C, the value of *D* was estimated to be about 4-fold higher on average than its value as measured in ROIs G and R, where the orientation of the diffusion gradients with respect to the lens tissue was very different. However, the values of *D* for the latter two ROIs were less consistent than those from the former two, and differed by about 8-fold. We believe that this inconsistency among the two cortical regions (G and R) could be explained by the different anterior and posterior curvatures of the lens. The shape of the bovine lens is such that its posterior radius of curvature is 1.5 times larger than its anterior (Kuszak et al., 2006). Hence, in the two cortical regions of G (anterior) and R (posterior), the local diffusion coefficients (*D*) will be different along the direction of the single applied gradient in this research. This phenomenon illustrates the need for a comprehensive multi-directional DTI protocol, in order to extract and meaningfully compare the *D* values of its different regions.

**Figure 6:**
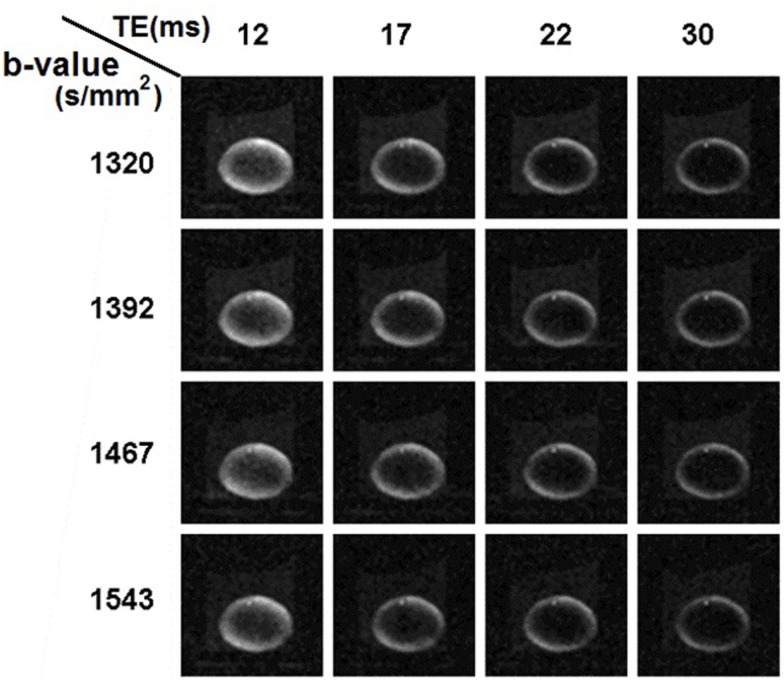
**Ocular lens imaged using diffusion weighting pulse sequences with combinations of different *b*-values and TEs**. In the second set of diffusion-weighted experiments (see Materials and Methods), *b*-values within the range of 1300–1600 s/mm^2^ were tested with a range of *T*_E_ from 12 to 30 ms in unidirectional diffusion-weighted scans. For the combinations tested, there was a pronounced, general loss of signal as *T*_E_ was increased; as well as attenuation as *b*-value was increased, consistent with the results shown earlier. The lens, image slice and diffusion gradients were oriented as in Figures 4 and 5.

In the second set of diffusion-weighted experiments, we tested the use of longer *T*_E_ values ranging up to 30 ms, at each of four different *b*-values selected from the 1300–1600 s/mm^2^ range that was indicated in the first set of experiments. In total, 16 combinations of *T*_E_ and *b*-value were tested (Figure 6). It was apparent from the images that as *T*_E_ was increased there was a pronounced general loss of signal; and that signal was also increasingly attenuated as *b*-value was increased.

**Figure 7:**
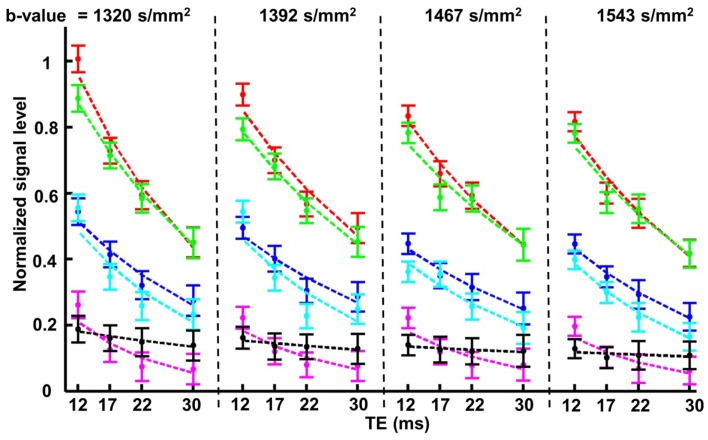
**The effects of varying *b*-value and *T*_E_, on the diffusion-weighted signal from the ocular lens**. In the second set of diffusion-weighted experiments (see Materials and Methods), four different *b*-values within the range of 1300–1600 s/mm^2^ were each tested with a range of *T*_E_ from 12 to 30 ms in unidirectional diffusion-weighted scans. The four *b*-values are represented by the four compartments in the figure above, separated by vertical dashed lines; in each compartment the four *T*_E_ values tested with the respective *b*-value are indicated. The colors of the data plots correspond to the six color-coded regions of interest (ROIs) indicated in Figure 5A. Graphs of the mean and standard error of the signal measured in the respective color-coded ROIs are shown for all 16 combinations of *b*-value and *T*_E_. The dashed lines represent exponential curves fitted to estimate the *T*_2_ values of various parts of the lens (see Text).

The results from this set of experiments were quantified following the approach used in Figure 5. The mean and standard error of the signal measured in each of the lens ROIs defined earlier were graphed as a function of increasing *T*_E_ and *b*-value (Figure 7). It was apparent from these graphs that for each of the *b*-values tested, the signal in the core of the lens fell below that of the bathing solution for *T*_E_ > 12 ms. At these *b*-values generally, the signal in the freely diffusing bathing solution would be at or very near the noise level in the images. As the signal in the core of the lens fell below that of the bathing solution, it therefore became difficult to accurately quantify the diffusion characteristics in the core. To ensure that the signal in the core remained above the noise in the images, and that the greatest signal differentiation between the ROIs around the cortex of the lens was obtained, a *T*_E_ of 12 ms and *b*-value of 1320 s/mm^2^ were chosen to be used in further experiments.

The data in Figure 7 also allowed estimates of the *T*_2_ of the ocular lens to be made. Exponential curves describing the signal decay as a function of *T*_E_ for a fixed *b*-value were fitted to the data from each ROI by adjusting *T*_2_ (Eq. 1) and the curves were plotted in Figure 7. The *T*_2_ was found to be (mean ± SE) 29.7 ± 1.2 ms for the lens cortex and 16.29 ± 1.9 ms for the lens core. These values were consistent with previous reports of short *T*_2_ in lenses of other species (Cheng et al., 1987; Wu et al., 1993).

**Figure 8:**
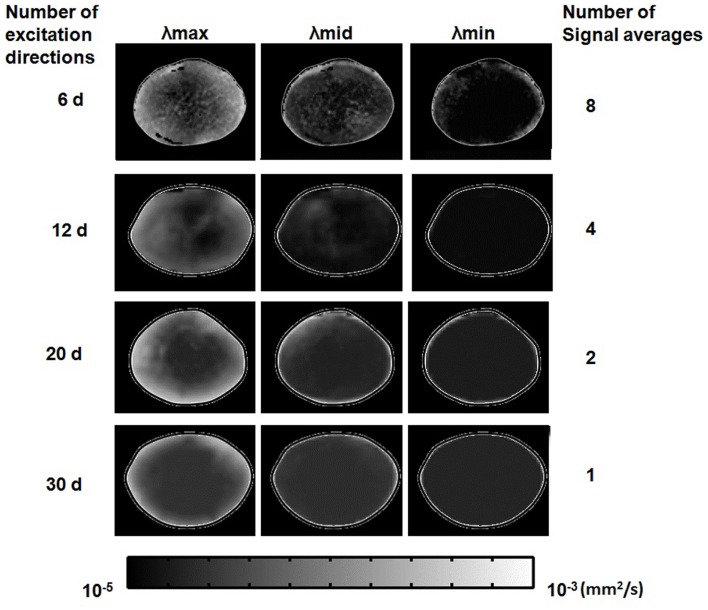
**Effects of the number of diffusion gradient directions, and signal averages, used in estimating the diffusion tensor**. Grayscale maps of λ_max_, λ_mid_, and λ_min_, passing through the visual axis of the lens (see Figures 1B,C), were calculated from lens image slices acquired using the four different scanning regimes described in Table 1 results from each regime are represented in one horizontal row of image maps shown above. In each row, one bovine lens has been scanned using a number of diffusion gradient directions (labeled at left) and a maximum corresponding number of signal averages (labeled at right) possible within the lens culture time limit (see Text). The eigenvalue maps are shown masked (manually) from the surround for ease of comparison. The very bright lens edge seen on some of the panels is due to an edge effect and thresholding.

After choosing the *b*-value and *T*_E_ above for diffusion-weighted imaging, we investigated the effects of using multiple diffusion gradient directions, and signal averaging, for DTI. The numbers of diffusion gradient directions and signal averages that could be used in combination in these experiments, were constrained by the maximum total imaging time allowed for cultured lenses (up to 12 h; see Materials and Methods). The scanning regimes used within this time limit are listed in Table 1. The effects of the different regimes on estimates of local diffusivity in the lens, can be observed in Figure 8, which shows maps of the maximum, minimum, and intermediate eigenvalues of the estimated diffusion tensor, calculated from lens image slices acquired under each regime. The overall map quality was greatly improved as the number of diffusion gradient directions was increased and (owing to the time constraint) the number of signal averages was decreased. It was clear from the eigenvalue maps that the effect of increasing the number of directions dominated over increasing the number of signal averages, in producing smoother, more radially symmetric eigenvalue maps.

The eigenvalues displayed in these maps, equivalent to ADCs, were of a similar order to the diffusion coefficients estimated earlier for the lens core and cortex from unidirectional scans (Figure 5). Relatively large maximum eigenvalues predominated at the lens periphery and these decreased steeply but smoothly (especially in the 30-direction image maps) going deeper into the lens. Smaller differences were observed for the intermediate and minimum eigenvalues as a function of depth in the lens. However, it should be noted that generally, a weak signal and hence a low SNR was observed in these experiments from the imaged lenses (Figure 8). At small SNR values, the eigenvalues of the diffusion tensor tend to diverge rapidly from their true values (Bastin et al., 1998). Such artificial deviations could lead to overestimation of the measured diffusion anisotropy in both homogeneous and inhomogeneous media. Such limitation in our methodology set the future direction of our research to improve the obtained SNR of our DTI experiments (e.g., by shortening *T*_E_), to enhance the reliability of our fractional anisotropy (FA) derivations.

**Figure 9:**
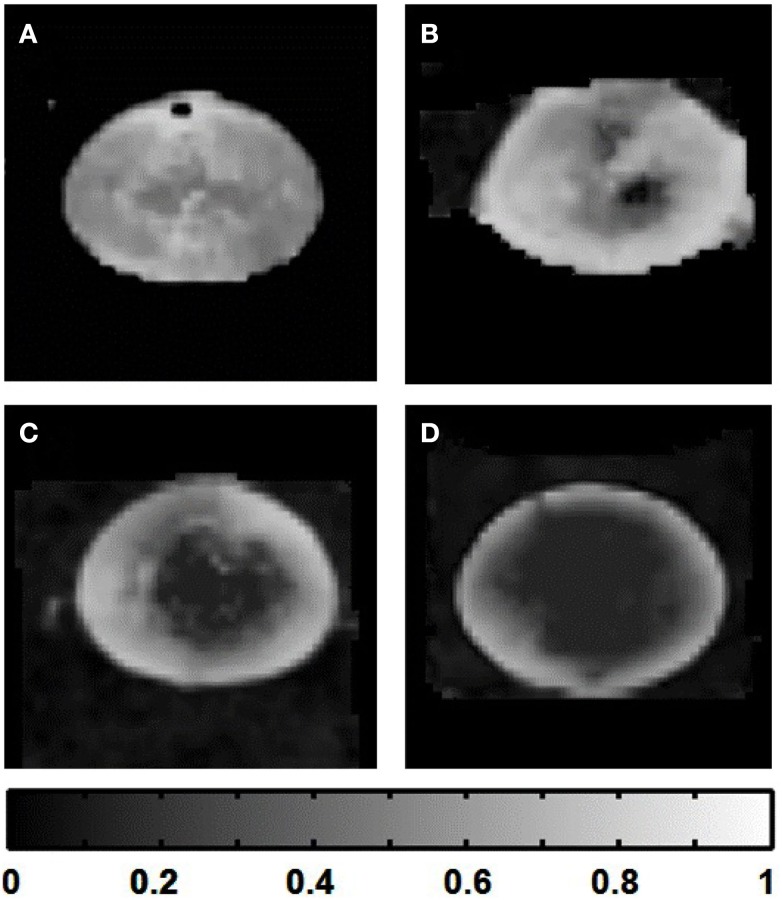
**Improvement of fractional anisotropy (FA) maps of the lens as the number of diffusion gradient directions was increased**. Grayscale FA maps passing through the visual axis of the lens (see Figures 1B,C) were calculated from eigenvalue maps (Figure 8) based on lens image slices acquired using the four different scanning regimes described in Table 1. Those regimes ranged from 6 to 30 diffusion gradient directions. **(A)** FA map calculated based on six diffusion gradient directions. **(B)** FA map based on 12 diffusion gradient directions. **(C)** 20 directions. **(D)** 30 directions. The grayscale strip at bottom indicates the FA value range in the images.

The performance of the different scanning regimes evaluated in Figure 8, in quantifying anisotropic diffusivity in the lens, was explored by using the eigenvalue maps to calculate maps of FA (Eq. 2; Le Bihan et al., 1992) across the lens (Figure 9).

(2) $$\text{FA}=\sqrt{\frac{3}{2}}\frac{\sqrt{{\left(\lambda_{1}-\hat{\lambda }\right)}^{2}+{\left(\lambda_{2}-\hat{\lambda }\right)}^{2}+{\left(\lambda_{3}-\hat{\lambda }\right)}^{2}}}{\sqrt{\lambda_{1}^{2}+\lambda_{2}^{2}+\lambda_{3}^{2}}}$$

where, λ is the calculated eigenvector and $\overset{\wedge }{\lambda }$ is the “trace”

λ^=(λ1+λ2+λ3)3

The FA maps produced showed much greater smoothness and radial symmetry as the number of scan directions was increased; while a more uniform pattern of decreasing anisotropy toward the lens core became apparent. These results suggested that given the time limit for our lens culture system (12 h), superior maps of FA could be obtained by applying diffusion gradients in 30 directions, with only 1 signal acquisition per direction (i.e., no averaging): this final protocol required a total scan time of ∼11 h.

## Discussion

Here we have reported our development of the DTI modality for the ocular lens. Various studies have demonstrated the potential of diffusion-weighted MRI for studying the whole eye (De Potter et al., 1995; Ettl et al., 2000) or specifically the ocular lens (Racz et al., 1983; Ahn et al., 1989; Lizak et al., 2000). In this paper, we systematically evaluated a number of regimes for performing DTI in the bovine ocular lens. Our final DTI protocol yielded significant new quantitative information on diffusivity in the lens, which was used to interpret previous studies of the lens and models for a lens micro-circulation system.

Since the ocular lens lacks a vascular system, its homeostasis depends on aqueous flow throughout the organ. However, despite its transparent appearance, the ocular lens is structurally and functionally heterogeneous (Mathias et al., 1981; Kuszak et al., 2004b). The geometry, functional coupling, and biochemistry of the fiber cells vary in different regions of the lens (Bassnett, 2002; Mathias et al., 2007), and water is more abundant and free to move in the outer cortex of the lens compared to its core (Gutsze et al., 1991). Such radial changes in water properties were supported by our initial development of *T*_2_-weighted imaging in the lens (Figure 3) where a steep gradient of signal was observed from the bright outer cortex to the dark lens center, corresponding to the known changes of water. The ocular lens consists overall of about 70% water and 30% other substances, the latter being predominantly the crystallin proteins (Paterson, 1972). In this kind of material *T*_2_ depends mainly on the local water:protein ratio, and the molecular state (bound or free) of the water (Cheng et al., 1987; Gutsze et al., 1991; Wu et al., 1993). Greater water content and freedom lead to longer relaxation times, which we observed in the outer cortex, consistent with earlier studies of lens water content.

The diffusivity of water in the ocular lens was investigated here by developing the DTI modality on the bovine lens. The capability of a pulse sequence to discriminate local variation of diffusion properties in a tissue depends on the diffusion weighting (Figure 2) of the pulse sequence, or *b*-value, according to Eq. 1: the higher the *b*-value, the greater the diffusion weighting. However, Eq. 1 also shows that very large *b*-values lead to generally low levels of signal. A series of *b*-values was tested on the lens (Figure 4): the resulting images showed the greatest attenuation due to diffusion (while remaining free from scanning artifacts) at *b*-values between 600 and 1600 s/mm^2^. In particular, the greatest differential attenuation between regions of the lens cortex where the direction of the diffusion gradients ran radially, vs. tangentially to the lens curvature, was achievable at *b*-values in the range of 1300–1600 s/mm^2^. This differential diffusive attenuation was presumably associated with the different orientations of the lens fiber cells with respect to the diffusion gradients. Subsequent tests of *b*-values within this range and *T*_E_ values from 12 to 30 ms showed that at *b* = 1320 s/mm^2^ and *T*_E_ = 12 ms the greatest signal differentiation in the cortex of the lens was obtained while ensuring that signal in the lens core remained above the noise. The choice of a relatively short *T*_E_ was consistent with the known, short *T*_2_ of the lens (Cheng et al., 1987; Wu et al., 1993).

The data showing signal as functions of *b*-value and *T*_E_ (Figures 5 and 7) allowed diffusion coefficients and values of *T*_2_ to be estimated for different lens regions using Eq. 1. The values of *D* where the direction of the diffusion gradients in the lens cortex ran tangentially to the lens curvature were on average 4-fold higher than locations where the direction of the diffusion gradients ran radially. This finding was of interest because it suggested anisotropic diffusivity in the lens cortex: the apparent diffusivity changed appreciably when the orientation of the diffusion gradients with respect to the underlying lens fiber cells changed. These calculated diffusion coefficients were found to be in agreement with other publications (Wu et al., 1993; rabbit), (Fischkoff and Vanderkooi, 1975; McNulty et al., 2004; oxygen diffusion into the bovine lens). Our findings are also comparable to a human DTI study (Moffat and Pope, 2002a) where *D* values are calculated to be two to threefold times higher in the cortex compare to the core. Furthermore, spatially averaged *D* of the young (healthy) human lens is found to be ∼0.22 × 10^−3^ (mm^2^.s^−1^) (Moffat et al., 1999) which is very close to our measurement of 0.22 × 10^−3^ (mm^2^.s^−1^) for the core of the bovine lens. It should be emphasized that the ocular lens of different species are not the same anatomically or physiologically and hence not directly comparable. Unfortunately, due to the very limited available literature on the diffusion parameters from the ocular lens, we are unable to comprehensively compare our results to other studies.

The core region did not indicate diffusive anisotropy, however the signal there was generally low, probably as a consequence of the low concentration of free water. The difference between the estimated values of *T*_2_ for the lens cortex and core was broadly consistent with previous estimates (Wu et al., 1993) and reflected the abundance of free water in the different regions as described earlier.

The elongated fiber cells of the ocular lens present significant structural anisotropy in the tissue (see Figure 1), (Kuszak, 1995). Therefore, in considering DTI it was especially important to explore the effects of applying the diffusion gradients in multiple directions, in order to reduce the directional dependence of the diffusion tensor estimates (Papadakis et al., 1999a; Jones, 2004). Our tests ranged from 6 to 30 directions, while acquiring as many signal averages as possible within the 12 h time frame of our lens culture system (Table 1). The resulting maps of diffusivity in the lens, in the form of eigenvalues representing ADCs in 3 dimensions (Figure 8), showed great improvement in quality as the number of directions was increased to 30, even as the number of signal averages was reduced to just a single acquisition per direction, to save scan time. More directions were not feasible on our hardware within the experimental time frame, however the maps produced using the 30 diffusion gradient directions showed relatively smooth and radially symmetric changes in the eigenvalues from the diffusion tensor, in maps running through the visual axis of the lens.

The improvement in the eigenvalue maps as the number of scan directions was increased had a great effect on estimates of FA in the lens. FA maps calculated from 6 up to 30 scan directions showed increasingly wide ranges of values, and a large, relatively isotropic central area of the lens appearing and becoming clearer as 30 directions were used (Figure 9). The maps also became much smoother and more radially symmetric as the number of directions was increased. These results suggested that the estimates of FA improved greatly even when increasing the number of diffusion gradient directions from 20 up to 30. Studies have suggested that up to 20 or even 30 directions can be required to make a robust estimate of the diffusion tensor (Jones, 2004). In addition, using multiple directions and multiple signal averages also improves measurements by increasing the effective SNR (Kaufman et al., 1989; Papadakis et al., 1999b; Le Bihan et al., 2001). The more uniform, symmetric FA pattern obtained here using 30 directions was consistent with the symmetric lens structure, and suggested that the necessary reduction of signal averages, in order to increase the number of diffusion gradient directions in our time-limited culture system, was worthwhile.

**Figure 10:**
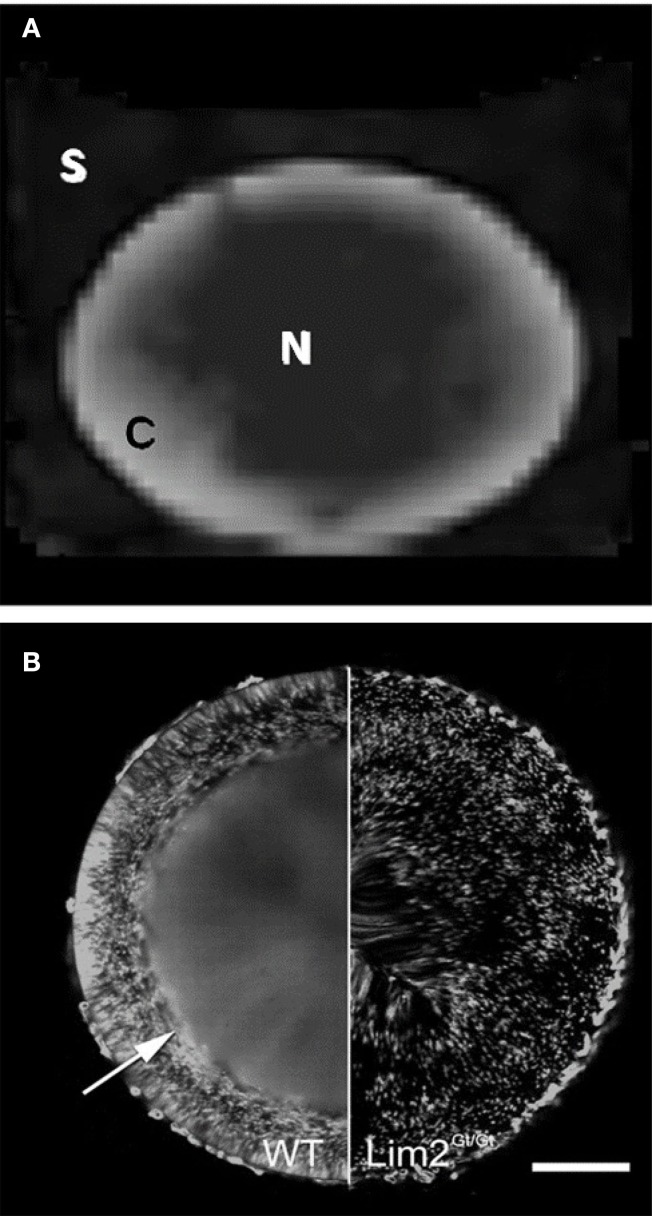
**Comparison of FA map with pattern of large molecule diffusion in the mouse lens**. **(A)** The FA map of the bovine lens from Figure 9D, showing anisotropy in the cortex and relative isotropy in the core. **(B)** Green fluorescent protein (GFP) expression in an otherwise normal (WT) mouse lens; and in a mouse lens deficient for the Lim2 protein (Lim2^Gt/Gt^), which is required in the formation of the large molecule diffusion pathway (LMDP; reproduced with permission from Shi et al. (2009). In the normal mouse lens the establishment of the LMDP allows GFP, made by scattered fiber cells, to diffuse evenly in the inner lens, in fibers located 100–200 μm below the lens surface *(arrow)*. In the Lim2-deficient lens the LMDP cannot form and GFP in the inner lens does not diffuse: it remains scattered in the cells that synthesize it. Scale bar, 250 μm.

The DTI methods we have developed here hold potential as new experimental inroads to understanding lens micro-circulation and homeostasis. For example, the FA map presented in Figure 9D shows an intriguing correspondence to a pattern of macromolecular diffusion revealed recently by different methods in the mouse lens (Shi et al., 2009; Figure 10). In the normal mouse lens, a large molecule diffusion pathway (LMDP) is believed to be established between fiber cells located 100–200 μm from the lens periphery. A “tripartite” model for the lens LMDP has been proposed (Shi et al., 2009) in which proteins in the outermost 50 μm of the mouse lens periphery are confined to the cells that synthesize them. Deeper in the lens cortex, where the cells have grown older, proteins diffuse between cells via the established LMDP in a predominantly circumferential direction (i.e., around the visual axis), achieving an even distribution in the tissue (Figure 10B). Near the center of the lens, proteins distribute between cells isotropically. Thus, there appear to be three distinct zones of macromolecular diffusion in the lens.

The LMDP, together with much smaller intercellular gap junction channels found in the lens, offers a mechanistic hypothesis to explain the FA map presented above. In previous studies we have shown that in the outer cortex of the rat lens, diffusion of small molecules via gap junction channels appears to be predominantly radial in direction (Jacobs et al., 2004). Since the LMDP works circumferentially in the lens cortex, the LMDP and gap junction channel pathways would be oriented orthogonally to each other there (Shi et al., 2009). If these pathways are also present in the bovine lens, the high FA values measured earlier at the lens periphery could simply result from the radial gap junction channel pathway predominating in the outermost layers of young fiber cells, while the LMDP is still being established. With greater depth into the lens, both (orthogonal) diffusion pathways working together could lead to a more isotropic distribution of water on the spatial scale sampled by our DTI measurements (encompassing thousands of fiber cells per voxel), causing FA to decrease. Finally, the low FA that we observed in the bovine lens core could reflect nearly isotropic diffusion of water occurring via both the LMDP pathway and gap junction channels. The latter have been associated with more isotropic cell–cell coupling in the deeper lens (Jacobs et al., 2004).

This straightforward explanation for our observed FA pattern in the lens could be evaluated by performing tracer experiments on directional cell–cell coupling in the bovine lens, to test the hypothesis that the relative contributions of the circumferential LMDP, and radial gap junction channel pathways, change as a function of increasing depth in the lens. Computational modeling of the lens circulation (Vaghefi et al., 2008, 2011) could be used to evaluate quantitatively whether the changing contributions of these pathways could account for the overall changes in FA that we have observed for water, across the lens. However, the variability of the ocular lens’s physiology and anatomy among different species should be taken into consideration before expanding on these conclusions. These dissimilarities include but are not limited to, the lens crystalline makeup and concentrations, age-related biochemical and biomechanical changes and ultra-structural (e.g., sutures formation) in different species (Bindels et al., 1983; Moffat et al., 1999; Moffat and Pope, 2002a; Kuszak et al., 2006). Hence, the applicability of the outcomes of this research should be taken into consideration alongside its limitations.

In conclusion, we have developed DTI in the bovine ocular lens. In testing a range of diffusion-weighted MRI regimes, we estimated values of *D* and *T*_2_ in different lens regions from unidirectional scans. We evaluated the effects of signal averaging and scanning the lens in multiple directions, and by DTI were able to produce smooth and radially symmetric maps of ADCs and FA across the lens, while keeping the total scan time within a viable lens culture system time frame. The ADC and FA maps produced by this study showed potential to inform current empirical findings on lens micro-circulation mechanisms, and suggested new hypotheses about diffusive pathways in the lens. The ability of the DTI modality to quantify aspects of lens micro-circulation non-invasively, with interrogation through to the lens center, makes it an attractive technique for future empirical studies.

## Conflict of Interest Statement

The authors declare that the research was conducted in the absence of any commercial or financial relationships that could be construed as a potential conflict of interest.
